# Supplementary material for: LSTM input timestep optimization using simulated annealing for wind power predictions
Source: PLoS One. 2022 Oct 7;17(10):e0275649. doi: 10.1371/journal.pone.0275649 (PMC9543974; doi:10.1371/journal.pone.0275649)
Supplement: Attachment — Submitted filename: Revision.doc [file pone.0275649.s001.doc]

**Revision Document**

**Paper ID:** PONE-D-22-05787

**Paper Title:** LSTM input timestep optimization using simulated annealing for wind power predictions

**I thank both reviewers for scrutinizing the manuscript and providing handy remarks to improve the manuscript.**

**Reviewer #1: After carefully reading and analysis of the paper, my comments as as follows**:

**1. The numerical method used in this paper should be investigated further using more statistical techniques.**

**We calculated the two-tailed p-values** between both results (without optimization algorithm and with simulated annealing) for all metrics and each wind farm. We used the following website to calculate the two-tailed P-values; this difference is considered very statistically significant by conventional criteria.

https://www.graphpad.com/quickcalcs/ttest2/

| **Evaluation Metrics** | **The two-tailed P value** |
| --- | --- |
| **MSE** | 0.0004 |
| **MAE** | 0.0004 |
| **RMSE** | 0.0008 |
| **r2\_score** | 0.0090 |
| **Explained\_variance\_score** | 0.0090 |

The response is added to the following location in the manuscript.

Page: 11

Line: 206

**2. I didn't find in-depth discussion around the computational cost of the proposed method.**

We added the following section, “**The computation cost of the proposed algorithm**,” to address this issue.

The response is added to the following location in the manuscript.

Page: 6

Line: 124 - 135

**3. The results should be discussed further at the end of the results sections to show the reasoning of the authors.**

The first interesting observation is reduced computation time. Consider the results for windfarm 1, table 4. The simulated annealing algorithm starts with lookback = 220, increasing the iterations. It mutates the lookback, rearranges the training data accordingly, and reports the evaluation metrics. The results for lookback = 236, shown in row 8, are the best, so we used that lookback to train the model for 500 epochs. Compare the same process with the simple LSTM, which iterates from lookback 1 to 500 and reports the epochs as shown in figure 8. The computation is the cost high in the simple LSTM.

Second, lookback for windfarms 1, 2, and 3 are different because the lookback is optimized for a particular wind farm. Third, we considered minimizing mean absolute error as an objective function. However, minimization of other parameters like mean explained variance or mean square error can also be considered an objective function.

The response is added to the following location in the manuscript.

Page: 11

Line: 210 - 220

**7. The results should be further explained to demonstrate that accurately proof/disproof the hypothesis.**

The computation cost of the whole process depends on the number of iterations of the simulated annealing. Consider the number of epochs = 30, lookback in the range [1-500], and 20 iterations of simulated annealing. For each iteration, the computational time will be different because it depends on the lookback. The max time for each iteration is within the range [Time algorithm takes for lookback 1 = T1 - Time algorithm takes for lookback 500 = T500]. The maximum time for 20 iterations can be 20*T500 if simulated annealing chooses lookback= 500 for all 20 iterations.

The time algorithm takes when lookback is linearly increased from 1 to 500 is the sum of all look back, which is greater than 20*T500. After finding the best lookback, the final step is to use LSTM with the best lookback for a specific number of epochs. The number of epochs in the final iteration is greater than the number of epochs used for the simulated annealing. So that is how simulated annealing reduces the execution time without reducing the performance.

The response is added to the following location in the manuscript.

Page: 11

Line: 218 - 235

**4. In terms of case-study, I wonder if authors can provide justifications on the suitability to stress test the proposed method.**

**The response is added to this section, “Stress analysis on lookback.”**

Thanks for highlighting this point.

When simulated annealing is used to find the optimal lookback, we specify a particular range in which simulated annealing should look for the next lookback. It directly affects the dataset size because it increases the lookback and results in the data replication.

Consider the following calculation to understand the following text,

The size of the dataset in memory is determined by the lookback based on the following formula.

**Lookback range = [0 – (Total Rows – 1)]**

**Total size in memory = (Total Rows – lookback)*(1+lookback)**

Consider total rows = 10

For lookback = 0; total size = (10 – 0)(1 + 0) = 10

For lookback = 1; total size = (10 – 1)(1 + 1) = 18

For lookback = 2; total size = (10 – 2)(1 + 2) = 24

For lookback = 3; total size = (10 – 3)(1 + 3) = 28

**For lookback = 4; total size = (10 – 4)(1 + 4) = 30**

**For lookback = 5; total size = (10 – 5)(1 + 5) = 30**

For lookback = 6; total size = (10 – 6)(1 + 6) = 28

For lookback = 7; total size = (10 – 7)(1 + 7) = 24

For lookback = 8; total size = (10 – 8)(1 + 8) = 18

For lookback = 9; total size = (10 – 9)(1 + 9) = 10

If we increase the lookback, the size of the dataset increase, so we cannot consider all the lookbacks when using simulated annealing. There must be a specific bound on lookback; otherwise, the system memory would not be able to handle it.

The response is added to the following location in the manuscript.

Page: 7

Line: 136 - 155

**5. I also would like the authors to discuss about two things around the optimisation method, overfitting and underfitting.**

You raised a good point regarding the optimization algorithm.

The overfitting and underfitting of simulated annealing can be inferred from the results. In the case of simulated annealing, the optimal lookback can be a sub-optimal solution, but whether the optimal/sub-optimal lookback overfits/underfits on the training or not can be inferred from the test MSE.

The test data is not used to find an optimal look back, so the LSTM performance without simulated annealing and with simulated annealing can be used to see whether the model overfits or not on the training data. So, we see that MSE on test data is reduced with simulated annealing, which means the model does not overfit the training data when simulated annealing is used to find an optimal lookback.

As far underfitting is concerned, the model does not underfit because when optimal lookback is used to train the model, the training MSE is reduced compared to when simulated annealing is not used.

The response is added to the following location in the manuscript.

Page: 9

Line: 193 - 204

**6. I also would like the authors to discuss about two things around the optimisation method, bias and model drift.**

We added one new section in the manuscript.

The optimization method is discussed in the **Simulated Annealing objective function** section

The response is added to the following location in the manuscript.

Page: 5

Line: 110 - 123

**8. Please highlight the hypothesis of this work in the text.**

We integrated simulated annealing (optimization algorithm) and LSTM (time series forecasting algorithm) to find an optimal lookback to reduce the computation time, find an optimal lookback, and improve the forecasting performance.

The proposed integration is also valid for other deep learning algorithms like BILSTM, GRU, and machine learning-based regression algorithms with minor modifications. It is also one of the future directions that we considered for optimization.

The response is added to the following location in the manuscript.

Page: 2

Line: 45 - 52

**Reviewer #2: The manuscript, in its present form, contains several weaknesses. Adequate revisions to the following points should be undertaken to justify the recommendation for publication.**

**1: please add future work to the conclusion section and discuss it briefly.**

We considered wind power prediction, and in the future, we plan to do it for other forecasting problems related to the energy sector like solar power prediction. Second, we considered only one optimization algorithm, simulated annealing, but optimization algorithms like particle swarm optimization and genetic algorithm can also be used to benchmark the performance. Benchmarking is necessary because different optimization algorithms yield different optimal lookbacks, and simulated annealing tries only a limited number of lookbacks (same as the number of iterations) to find an optimal one. Third, we considered only the lookback parameter for optimization, but simulated annealing can also be used to optimize multiple parameters like the number of epochs, which will undoubtedly increase the overall computation cost. The LSTM can be replaced with BILSTM, GRU, and machine learning-based regression algorithms with minor modifications.

The response is added to the following location in the manuscript.

Line: 249 - 259

**2. The contribution is not stated also add at the end of the introduction section.**

Following are the scientific contribution of this research work.

We integrated simulated annealing (optimization algorithm) and LSTM (time series forecasting algorithm) to find an optimal lookback to reduce the computation time, find an optimal lookback, and improve the forecasting performance.

The proposed integration is also valid for other deep learning algorithms like BILSTM, GRU, and machine learning-based regression algorithms with minor modifications. It is also one of the future directions that we considered for optimization.

The response is added to the following location in the manuscript.

Page: 2

Line: 45 - 52

**3. What is the main reason for choosing the simulated annealing algorithm?**

This work extends our previously proposed algorithm, which used a genetic algorithm with LSTM for time step optimization. In that algorithm, we optimized the lookback and the number of neurons in each layer, which took a lot of time. The focus of that work was on improving the performance but not reducing the computation time, so we decided to use simulated annealing to improve the performance (lookback) and reduce the computation time. The success of a genetic algorithm depends on the number of generations and the number of instances in each generation. LSTM has to be executed 100 times for ten generations and ten instances, whereas the simulated annealing requires only a small number of iterations to find an optimal lookback.

The response is added to the following location in the manuscript.

**Line: 86 - 94**

**4. The results and discussion section has to be improved, where more details of the achieved results should be stated clearly in this section. In addition, authors also have to provide some insight discussion of the results.**

This section discusses the results generated by all two algorithms: \texttt{Simple LSTM} (A simple lstm model with lookback = 1-500 and epochs = 30), and \texttt{LSTM with Simulated Annealing} (Lstm model with 20 iterations of simulated annealing, 30 epochs, and lookback = 1-500).

The first interesting observation is reduced computation time. Consider the results for windfarm 1, table 4. The simulated annealing algorithm starts with lookback = 220, increasing the iterations. It mutates the lookback, rearranges the training data accordingly, and reports the evaluation metrics. The results for lookback = 236, shown in row 8, are the best, so we used that lookback to train the model for 500 epochs. Compare the same process with the simple LSTM, which iterates from lookback 1 to 500 and reports the epochs as shown in the figure 8. The computation is the cost high in the simple LSTM.

Second, lookback for windfarms 1, 2, and 3 are different because the lookback is optimized for a particular windfarm. Third, we considered minimizing mean absolute error as an objective function. However, minimization of other parameters like mean explained variance or mean square error can also be considered an objective function.

The response is added to the following location in the manuscript.

Page: 11

**Line: 210 - 220**

**5. Please explain the objective function mathematically in a separate section.**

We added the section “**Simulated Annealing objective function**” in the manuscript explaining the objective function.

The response is added to the following location in the manuscript.

Page: 5

Line: 110 - 123
